# Commentary: Fu's subcutaneous needling for knee osteoarthritis: a systematic review and meta-analysis

**DOI:** 10.3389/fmed.2025.1693393

**Published:** 2025-09-25

**Authors:** Doori Kim, Yoon Jae Lee, In-Hyuk Ha

**Affiliations:** Jaseng Spine and Joint Research Institute, Jaseng Medical Foundation, Seoul, Republic of Korea

**Keywords:** Fu's subcutaneous needling (FSN), acupuncture, knee osteoarthritis, efficacy rate, commentary

## Introduction

We read with great interest the recent article by Zhao et al. (1) entitled “*Fu's subcutaneous needling for knee osteoarthritis: a systematic review and meta-analysis*.” Knee osteoarthritis (KOA) underlies a major global health burden that substantially impairs quality of life and incurs high medical and socioeconomic costs (2, 3). Investigations of non-pharmacological strategies, especially novel acupuncture-related therapies including Fu's subcutaneous needling (FSN), are meaningful and timely. The authors are commended for synthesizing the available evidence to analyze this relatively new intervention.

## Current challenges facing FSN research and practice

While this meta-analysis presents intriguing clinical findings, several methodological issues warrant further consideration. FSN is not yet internationally standardized, and neither major international guidelines for KOA management nor widely recognized clinical practice frameworks currently cover FSN. Although FSN has been actively investigated in China for KOA and various other musculoskeletal disorders, most international readers remain unfamiliar with this technique, its clinical adoption, and its procedural details. A more thorough introduction to FSN would thus enhance the accessibility of this review.

Moreover, the technical parameters of FSN—such as needle type, insertion depth, sweeping frequency, flushing procedure, and catheter retention—remain poorly described and lack widespread standardization in practice. This heterogeneity complicates pooling of results and limits the interpretability and generalizability of the findings. Explicitly addressing these uncertainties would strengthen the clinical and international relevance of this review. Moving forward, collaboration with international academic societies and efforts toward procedural standardization will be crucial for enhancing the credibility, reproducibility, and global applicability of FSN research.

## Outcome choice and interpretability

A central concern relates to the choice of primary outcome. The authors designated “total efficacy rate” as the main endpoint. However, the definition of “efficacy” varies across individual studies, raising questions about the validity of combining such outcomes in a meta-analysis. More importantly, this composite indicator has not been internationally recognized in musculoskeletal research.

According to the Methodological Expectations of Cochrane Intervention Reviews standards (4), the assessed outcomes should be those that are important or critical to patients. The number of outcomes should be limited to avoid selective reporting. Moreover, the use of core outcome sets (COS) is highly recommended. According to the updated Outcome Measures in Rheumatology-Osteoarthritis Research Society International (OMERACT–OARSI) core domain set (5), clinical trials on knee osteoarthritis should at minimum report pain, physical function, quality of life, patient global assessment, and adverse events. In this context, validated and widely accepted measures, such as the Western Ontario and MacMaster Universities Osteoarthritis Index score, visual analog scale for pain, or numeric rating scale for pain should have been prioritized as primary outcomes.

The use of a non-standard composite measure like “total efficacy rate” in Zhao et al.'s FSN meta-analysis contrasts with internationally endorsed core outcome frameworks, reducing its comparability and clinical interpretability.

## Discussion and future directions

This meta-analysis highlights the potential benefits of FSN in KOA, particularly in pain relief, functional improvement, and modulation of inflammatory markers. Nevertheless, uncertainties regarding the methodological standardization of FSN and the appropriateness of outcome selection substantially limit the strength of the conclusions. Future research should (1) adopt internationally validated outcomes aligned with COS frameworks, (2) establish consensus-based procedural standards for FSN, and (3) extend clinical trials beyond China through multinational collaborations. These steps would substantially enhance the robustness, reproducibility, and global relevance of research into FSN for KOA management.
